# Investigating the impact of metacognitive regulation on students’ academic performance: a quantitative study of Chinese distance learners

**DOI:** 10.3389/fpsyg.2026.1726956

**Published:** 2026-02-18

**Authors:** Yanxing Xue, Fariza Khalid

**Affiliations:** Faculty of Education, Universiti Kebangsaan Malaysia (UKM), Bangi, Malaysia

**Keywords:** academic performance, distance education, metacognitive regulation, metacognitive monitoring, self-efficacy

## Abstract

Distance learners are required to manage their learning processes independently, yet empirical evidence on how different components of metacognitive regulation relate to academic performance remains limited in the Chinese distance education context. This study examines the roles of planning, monitoring, regulation, and self-efficacy, as well as the mediating role of task strategies. A quantitative survey was conducted with 381 Chinese distance learners. Data were analyzed using PLS-SEM to test direct and mediated relationships among metacognitive regulation components, task strategies, and perceived academic performance. Metacognitive monitoring, regulation, and self-efficacy showed significant positive associations with academic performance, while planning showed only a marginal effect. Task strategies were positively related to performance and were significantly predicted by all metacognitive components, supporting a mediated pathway from regulation processes to learning outcomes. The findings indicate that monitoring accuracy, adaptive regulation, and self-efficacy are key factors linked to performance in distance learning, partly through their support of effective task strategies. The results highlight the importance of strengthening metacognitive regulation capacities in online and distance education settings.

## 1 Introduction

Contemporary learning environments, especially those mediated by online and distance modes, require learners to take greater responsibility for directing and evaluating their own learning. Their educational success is highly dependent on their interaction with the efficient use of strategies, including those of metacognitive strategies, which allow them to control the learning process (Vosniadou et al., 2021). The poorly organized knowledge regarding metacognitive regulation and its influence on student support hampers their overall success, specifically within the distance education environment. Prior work suggests that metacognition is closely tied to academic achievement because it supports purposeful planning, ongoing monitoring, and adaptive regulation of learning activities (Samuel and Okonkwo, 2021). Yet, it is still unclear which metacognitive facets are most strongly associated with academic performance when learners’ self-efficacy and task strategies are considered simultaneously.

In distance education, self-efficacy functions as a key motivational resource that supports persistence under academic challenge (Celik, 2022). Lower self-efficacy is often accompanied by heightened anxiety and diminished perceived control, which may reduce sustained engagement and performance. Moreover, within the self-regulated learning framework, inadequate metacognitive regulation can hinder learners’ ability to clarify task demands, deploy effective strategies, and monitor progress (Roberts, 2021). Prior evidence further suggests that limited metacognitive regulation is associated with weaker academic outcomes in distance learning settings, where learners depend heavily on planning and monitoring to complete demanding tasks (Anthonysamy, 2021).

The study highlights the benefits that metacognition regulation can provide to distance learners in China, in terms of developing their academic performance. The findings would contribute toward enhancing the concepts of the metacognition cyclic phase model that emphasize the importance of such strategies benefiting learner performance. The findings would further develop the theoretical concepts of self-efficacy, planning, regulating and monitoring and their benefits toward academic performance in distance learning. Apart from this, the findings would also prove to be helpful for education policymakers, specifically for institutes focusing on distance education; the developments and areas of focus regarding distance education would be highlighted. This would enhance the academic performance of all students enrolled, irrespective of their learning patterns.

Hence, the study aims to analyze how the implemented metacognitive regulation in distance education can benefit the academic performance of Chinese distance learners. The following objectives are aimed to be satisfied through the research: (a) to analyze the impact of self-efficacy on academic performance of Chinese Distance learners; (b) to determine the role of planning in enhancing academic performance of Chinese Distance learners; (c) to examine the influence of regulations on the development of academic performance of Chinese Distance learners; (d) To analyze the impact of monitoring in enhancing academic performance of Chinese Distance learners; (e) to identify the role of task strategies in developing the usage of metacognition factors like planning, monitoring and regulation for better academic performance of Chinese Distance learners.

## 2 Theoretical perspective

Grounded in seminal work, metacognition was first articulated by Flavell as “cognition about cognition” and elaborated as a system of metacognitive knowledge and metacognitive experience that supports goal attainment (Flavell, 1979). Complementing this view, Brown emphasized that effective metacognitive regulation depends on executive control and the deliberate use of cognitive knowledge to manage cognition and performance (Brown, 1978). From a social-cognitive perspective, Schunk further argued that learners’ self-efficacy beliefs shape motivation, strategy selection, and persistence, thereby influencing how strongly students engage in metacognitive regulation during learning (Schunk, 1989; Schunk, 1991). Building on these foundational perspectives, the present study adopts a cyclical metacognitive model to examine how self-efficacy and metacognitive processes relate to academic performance among Chinese distance learners.

### 2.1 Cyclical phases model of metacognition

The metacognitive cycle is commonly described as a cyclical self-regulated learning (SRL) process that can enhance learners’ self-awareness during learning. This model is often attributed to Zimmerman and emphasizes SRL as a structured sequence of goal setting, performance monitoring, and self-reflection (Anthonysamy, 2021). Specifically, it comprises three phases: forethought, performance, and self-reflection.

**Figure 1:**
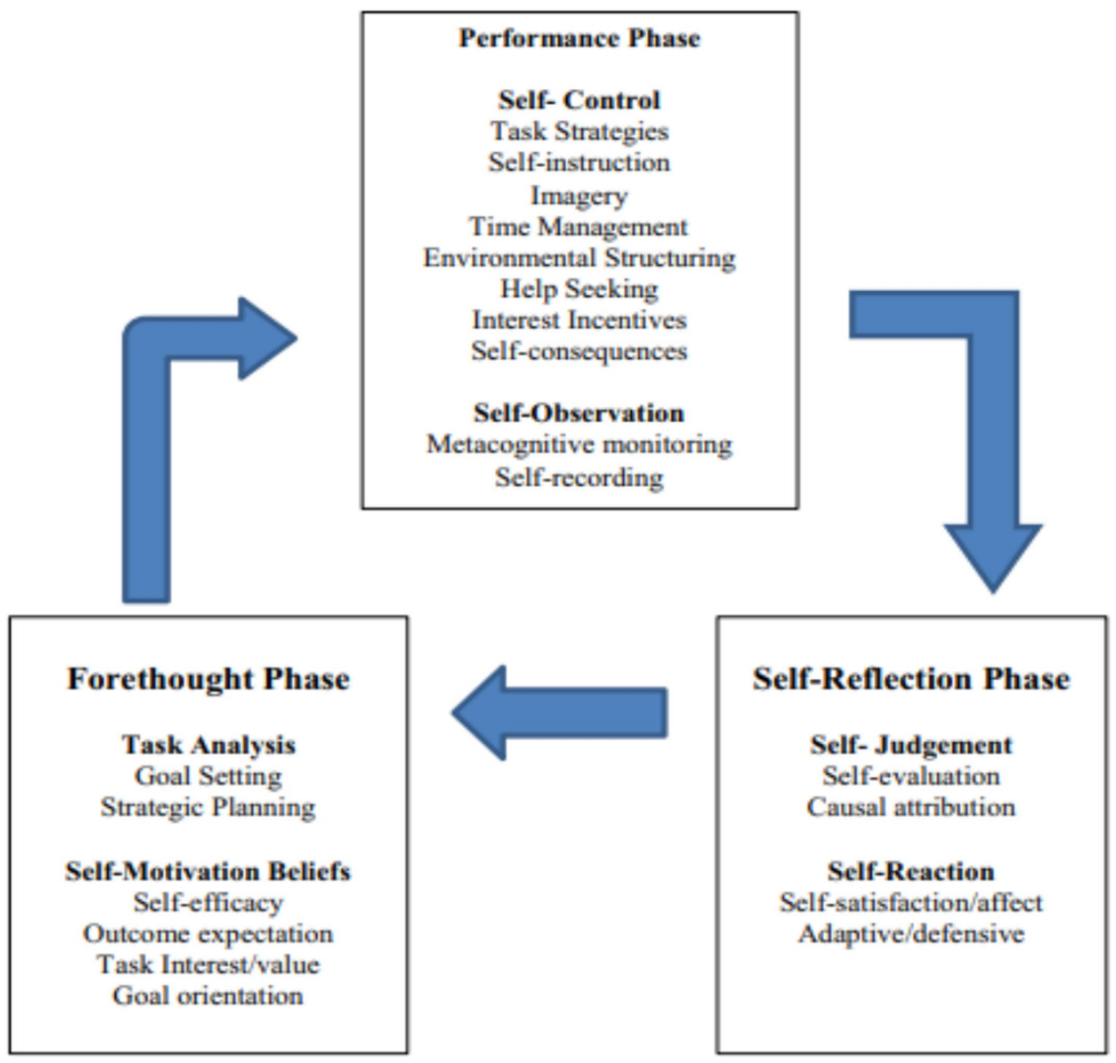
Cyclical phases model of self-regulated learning (SRL). Adapted from Zimmerman (2002), as reproduced in Anthonysamy (2021).

Grounded in Zimmerman’s cyclical model of self-regulated learning, Figure 1 conceptualizes learning as an iterative process across forethought, performance, and self-reflection phases (Zimmerman, 2002; Panadero, 2017). In the forethought phase, learners conduct task analysis (e.g., goal setting and strategic planning) while motivational beliefs, particularly self-efficacy, shape the level of effort, persistence, and the likelihood of initiating effective strategies (Zimmerman, 2002; Schunk, 1991; Panadero, 2017). In the performance phase, learners implement task strategies and enact self-control and self-observation, where metacognitive monitoring provides real-time information about comprehension and progress and supports regulatory adjustments (e.g., time management, help seeking, and strategy refinement) (Flavell, 1979; Zimmerman, 2002; Panadero, 2017). In the self-reflection phase, learners evaluate outcomes (self-judgment) and respond effectively and behaviorally (self-reaction), generating feedback that informs subsequent planning and regulation in the next cycle (Zimmerman, 2002; Panadero, 2017).

For the present study, this SRL cycle provides a clear theoretical lens to distinguish metacognitive regulation processes (planning, monitoring, and control/regulation of cognition) from motivational beliefs such as self-efficacy, and to explain how these mechanisms jointly relate to distance learners’ academic performance. Empirically, evidence from online learning contexts suggests that metacognitive regulation (planning, monitoring, and regulation) are reliable predictors of learning performance, underscoring the relevance of examining metacognitive regulation as a pathway to academic performance in ODL settings.

Although planning, monitoring, and regulation are closely related facets of metacognitive regulation, they represent conceptually distinct processes that become most salient at different phases of the self-regulated learning cycle. In Zimmerman’s cyclical model, planning is primarily situated in the forethought phase (e.g., goal setting and strategic preparation), monitoring reflects real-time self-observation during task performance, and regulation involves strategic control and adjustment in response to monitoring feedback (Zimmerman, 2000). A similar distinction is evident in Nelson and Narens’ framework, which separates monitoring (evaluative awareness) from control (regulatory action) as two core functions of metacognitive regulation (Nelson and Narens, 1990). Making these boundaries explicit helps justify why the three constructs should be related yet non-redundant, and why they may show different links with task strategies and academic performance in distance learning contexts.

### 2.2 Impact of self-efficacy on academic performance

Ivanova and Kafadarova (2023) suggested that self-efficacy may be particularly consequential in distance learning, where learners often rely more on internal motivation and self-management, while it remains an important resource in traditional face-to-face settings. Building on social cognitive theory, Pajares (2003) emphasized that self-efficacy beliefs shape how learners approach academic challenges by influencing effort, persistence, and the strategic regulation of learning. From this perspective, self-efficacy is not merely an outcome belief; it functions as a motivational driver that supports sustained engagement and effective strategy use, thereby contributing to academic performance. Consistent with this view, prior studies have generally reported positive associations between self-efficacy and academic performance among distance learners, particularly in online learning environments (Celik, 2022). Moreover, learners’ self-efficacy may also be strengthened through goal orientation, effort regulation, and deep processing strategies, which can reinforce confidence in managing academic tasks (Navarro et al., 2023). Nevertheless, the magnitude of these relationships may vary across contexts, as cultural factors and the timing of measurement can influence how learners interpret and report their academic self-efficacy.

Learners’ attitudes toward and familiarity with online instruction have been shown to shape their self-efficacy in distance learning settings (De Backer et al., 2022). Stronger self-efficacy may, in turn, be associated with higher perceived task value, which can support learners’ engagement in metacognitive regulation during learning (Sotardi, 2022). Taken together, prior research broadly suggests that both self-efficacy and metacognition are related to academic performance. Self-efficacy reflects learners’ confidence in their capability to succeed, whereas metacognition involves monitoring understanding and regulating strategies as learning unfolds. These complementary resources may help learners sustain effort and make more adaptive study decisions, thereby supporting stronger academic outcomes.

### 2.3 Role of planning on academic performance

Roberts (2021) emphasized that metacognition and effective planning are important for supporting academic performance because they help learners clarify task demands, monitor progress, and adapt their learning approaches over time. In particular, metacognitive regulation enables learners to track their understanding and make strategic adjustments during learning, which can contribute to better outcomes (Stanton et al., 2021). For example, learners may set clear and attainable goals to sustain motivation and focus their effort. Through planning, they can also identify appropriate resources and select strategies that fit the task requirements, which is especially valuable in distance learning contexts where external guidance may be limited (Vosniadou et al., 2021). In addition, planning can support more informed resource selection by encouraging learners to reflect on their strengths and weaknesses before engaging with learning tasks.

Time management is a key component of planning in distance learning because it can reduce procrastination, support effective time allocation, and ensure adequate preparation (Chang et al., 2021). When preparing for online learning tasks, learners may plan activities such as practising problems, reviewing notes, or creating flashcards to structure their study process (Samuel and Okonkwo, 2021). During distance learning, they can also plan to summarize key concepts through self-questioning as a way to maintain focus and monitor understanding. With a clearer plan in place, learners are better positioned to identify gaps in their learning and adjust their metacognitive regulation accordingly. Overall, prior studies suggest that incorporating planning as part of metacognitive regulation can support learners’ academic performance.

### 2.4 Significance of monitoring on academic performance

Guo (2022) noted that metacognition involves monitoring processes through which learners evaluate their understanding and learning progress. Such monitoring helps students make sense of how they are learning and supports strategic adjustments, thereby fostering more efficient learning. Sustained metacognitive monitoring can encourage learners to adopt and refine task-appropriate strategies that support academic performance (Taouki et al., 2022). By repeatedly checking their progress and strategy use, students may also become more aware of what they are doing during learning and why certain approaches work better than others. This awareness can strengthen learners’ sense of control and, in turn, support self-efficacy and motivation—both of which are generally associated with academic performance. In distance learning contexts, where access to immediate face-to-face guidance may be limited (Samuel and Okonkwo, 2021), metacognitive monitoring can help students identify weaknesses, recognize when support is needed, and decide when to seek help or adjust their learning strategies.

Stanton et al. (2021) reported that active monitoring can improve the depth and accuracy of learning. In the context of metacognitive regulation, monitoring can be enacted through concrete practices such as asking clarifying questions, checking understanding during learning, and reflecting on one’s own learning process (Van Loon and Oeri, 2023). For distance learners, these monitoring activities can support more accurate judgments of what is understood versus what remains unclear, which in turn facilitates more targeted strategy adjustment and better retention. As a result, metacognitive monitoring is often found to be significantly associated with academic performance.

### 2.5 Influence of regulating academic performance

Metacognition refers to learners’ awareness of their own cognition and their ability to regulate cognitive activity, both of which are important for academic performance (Shao et al., 2023). Within this construct, metacognitive regulation typically involves planning, monitoring, and evaluating or adjusting learning strategies, which supports more accurate self-regulated learning. In distance learning contexts, where learners have fewer immediate opportunities for face-to-face guidance, self-regulation is particularly important for sustaining progress and achieving stronger academic performance (Pucillo and Perez, 2023). Conceptually, metacognitive regulation works alongside metacognitive knowledge: learners draw on what they know about tasks, strategies, and themselves to plan and monitor learning, and then adapt their approach based on feedback. In this way, stronger metacognitive knowledge and regulation may jointly support more effective learning and better academic outcomes among distance learners.

Cheng et al. (2025) noted that regulation enables learners to evaluate the effectiveness of their learning methods and to make timely adjustments to their approach. Such regulatory activity is therefore a key metacognitive component in distance learning, where learners must manage tasks with greater autonomy (Stanton et al., 2021). By taking ownership of their learning, students can approach academic problems more strategically, select more suitable strategies, and refine them based on feedback. In turn, regulation may support stronger academic performance among distance learners.

### 2.6 Mediating role of task strategies on the relationship between self-efficacy, planning, monitoring, regulation, and academic performance

Khan (2023) suggested that instructors can shape students’ self-efficacy through the design of learning tasks and the standards embedded in those tasks. When tasks are structured to support metacognitive regulation, for example, by prompting planning, monitoring, and strategy adjustment, students may develop stronger confidence in managing academic challenges and meeting course expectations. Similarly, Zheng et al. (2021) noted that instructional approaches and task strategies implemented in courses can provide conditions that foster students’ self-efficacy. In this sense, supportive task design may help learners cope more proactively with academic demands by encouraging deliberate self-management and adaptive responses to stressors.

Acosta-Gonzaga (2023) has further highlighted how motivation influences the metacognitive regulation of students toward academic growth. Motivated students promote cognitive processes that allow to develop a higher level of self-efficacy and intrinsic motivation (Usán Supervía and Quílez Robres, 2021). The tasks provided to the students focusing on metacognitive regulation are beneficial in allowing students to have control over their actions to achieve higher academic performance.

According to Bernacki et al. (2021), students who have utilized the required resources, like supported planning and cognitive strategies, have outperformed those who have lacked such implementation. This evidence underscores the importance of planning and task strategies for learning success in distance education. In line with this, education modules have been developed to promote planned and organized study efforts and to strengthen strategy use for improved academic performance (Rajabalee and Santally, 2021). In such settings, task strategies can encourage learners to take a more proactive role in planning and to manage academic challenges more effectively.

Metacognition can help learners develop more expert-like thinking and engage in learning activities more efficiently (Stanton et al., 2021). Through metacognitive awareness, students can reflect on which strategies they are using, plan their study approach, and monitor whether those strategies are working. As learning progresses, they can evaluate task strategies against their goals and outcomes and adjust when necessary (Markandan et al., 2022). Consequently, learners with stronger metacognition are better able to detect concepts they do not yet understand and to select more suitable strategies to address those gaps.

Monitoring activities are an integral part of metacognitive regulation and are often linked to stronger academic performance. In particular, students’ self-monitoring helps them keep track of their understanding and progress, which can support more timely adjustments during learning (Van Loon and Oeri, 2023). Through monitoring, learners can evaluate whether a chosen strategy is effective and, where needed, implement alternative strategies in their study routines to better align their actions with academic goals (Mahmud and German, 2021). In this way, monitoring supports more purposeful strategy use and sustained progress toward learning outcomes.

Shao et al. (2023) noted that effective regulation of learners’ behavior and cognition can support stronger academic performance. In distance education, this regulatory capacity is often enacted through purposeful use of task strategies and collaborative engagement in goal-directed activities, which can help learners stay aligned with shared learning objectives (Xu et al., 2022). More broadly, self-regulated learning approaches provide learners with a structured means of managing the learning process across distinct phases, supporting greater control over goal setting, performance monitoring, and reflection (Öztürk and Çakıroğlu, 2021). Accordingly, even in remote learning contexts, students can analyze task requirements and translate them into concrete steps and strategies to enhance their chances of success.

## 3 Conceptual framework and hypothesis statement

### 3.1 Conceptual framework

**Figure 2:**
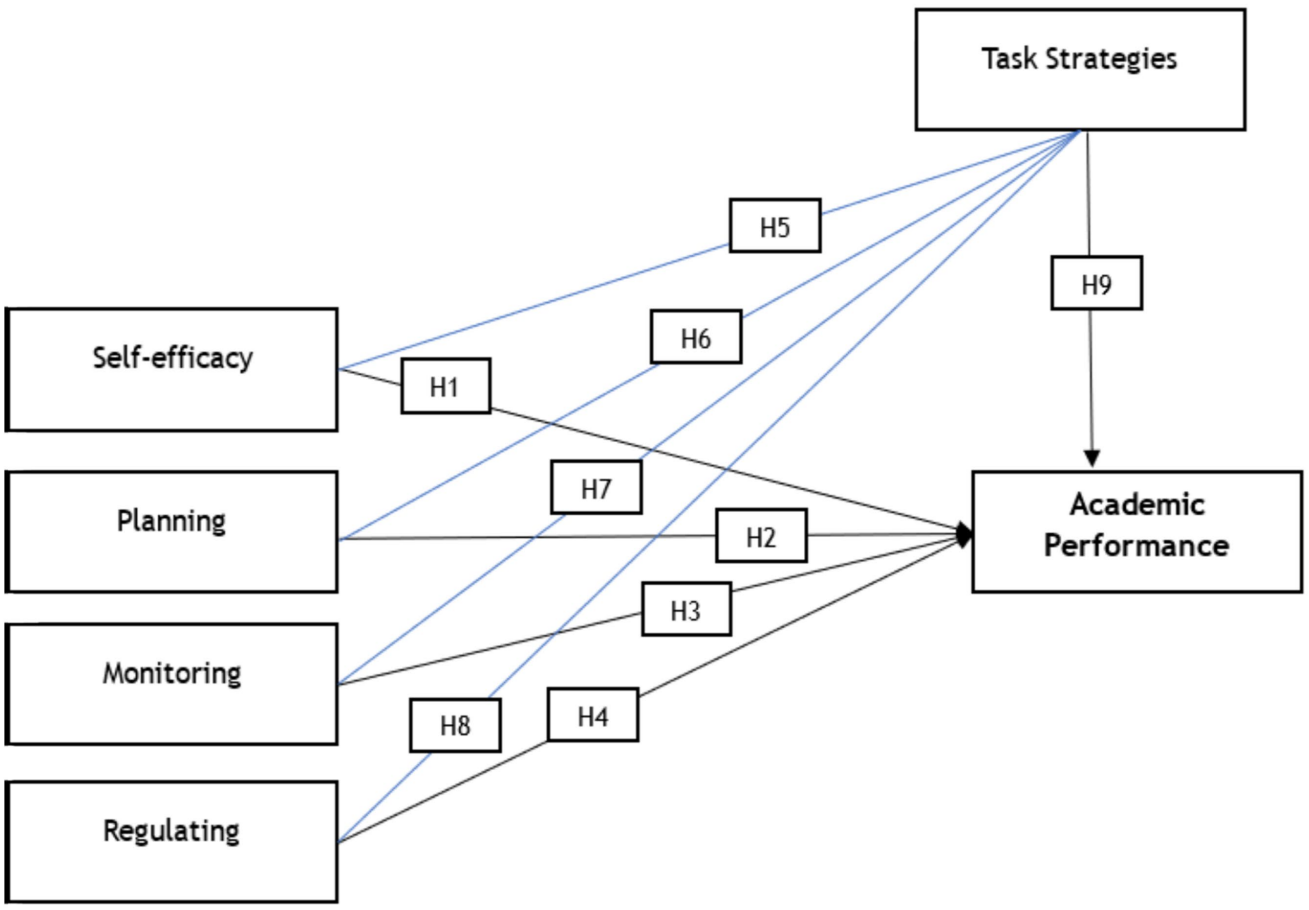
Conceptual framework.

Figure 2 shows the relationship among the research variables, which form the following hypothesis statements:

### 3.2 Hypothesis statement

From a social-cognitive perspective, self-efficacy shapes learners’ goal setting, effort investment, persistence, and strategy selection, thereby enhancing academic achievement (Bandura, 1997; Zimmerman, 2000). Meta-analytic evidence with university samples further shows a moderate-to-strong positive association between self-efficacy and academic performance (Richardson et al., 2012; Honicke and Broadbent, 2016). Accordingly, the present study hypothesizes a positive relationship between self-efficacy and academic performance (H1).

*H1*: Self-efficacy is positively associated with academic performance among Chinese distance learners.

In self-regulated learning frameworks, the forethought or planning phase is conceived as the starting point of effective learning, encompassing goal decomposition, time management, and resource allocation (Winne and Hadwin, 1998; Zimmerman, 2000). Systematic reviews in online higher education further indicate that planning-oriented strategies, particularly goal setting and time management, are significantly associated with academic achievement (Broadbent and Poon, 2015). Accordingly, the present study proposes that planning is positively related to academic performance (H2).

*H2*: Planning is positively associated with academic performance among Chinese distance learners.

Monitoring enables learners to inspect comprehension and progress in real time, detect deviations, and initiate corrective adjustments (Winne and Hadwin, 1998). Metacognitive monitoring shows a consistent positive association with academic achievement in university samples and is especially consequential in online and distance contexts where immediate instructor oversight is limited (Broadbent and Poon, 2015). Accordingly, the present study proposes the following hypothesis (3):

*H3*: Monitoring is positively associated with academic performance among Chinese distance learners.

Regulation refers to adjusting strategies and effort in response to monitoring feedback (e.g., shifting the level of processing, strengthening effort control), functioning as the error-correction phase within the SRL cycle (Zimmerman, 2000; Panadero, 2017). Empirical evidence indicates that metacognitive self-regulation is significantly associated with academic achievement, with effects particularly pronounced in online learning environments (Broadbent and Poon, 2015; Nelson and Narens, 1990). Accordingly, the present study proposes the following hypothesis (4):

*H4*: Regulating is positively associated with academic performance among Chinese distance learners.

The motivational pathway to strategy use posits that self-efficacy, by strengthening perceived value and control, promotes the adoption of deep-processing strategies such as elaboration and organization as well as greater persistence (Pintrich and De Groot, 1990; Bandura, 1997). Reviews and meta-analyses likewise identify self-efficacy as a strong predictor of learning strategy use (Richardson et al., 2012; Honicke and Broadbent, 2016). Accordingly, the present study proposes the following hypothesis (5):

*H5*: Self-efficacy is positively associated with task strategies among Chinese distance learners.

Within the Winne and Hadwin model, the planning phase maps goals and task features onto a repertoire of concrete strategies, deciding which strategies to use, in what sequence, and for how long, thereby increasing the specificity and fit of strategy selection (Winne and Hadwin, 1998). Studies of online learning similarly find that sound planning co-occurs with more frequent use of task strategies (Broadbent and Poon, 2015; Barnard et al., 2009). Accordingly, the present study proposes the following hypothesis (6):

*H6*: Planning is positively associated with task strategies among Chinese distance learners.

Improving the accuracy of metacognitive monitoring was linked to learners’ task-strategy selection. In an experiment on text learning, students whose monitoring was enhanced (via procedures that increased the accuracy of their judgments of learning) allocated study time more adaptively and selected more appropriate restudy materials, which in turn yielded better comprehension outcomes, direct evidence that monitoring drives subsequent strategy use. This mechanism maps onto distance learning, where learners must self-select tactics in the absence of immediate instructor oversight (Thiede et al., 2003). Accordingly, the present study proposes the following hypothesis (7):

*H7*: Monitoring is positively associated with task strategies among Chinese distance learners.

Regulation is the process of converting monitoring feedback into specific strategic adjustments and effort regulation (e.g., shifting from surface to deep processing, increasing self-testing and self-explanation) (Zimmerman, 2000). The covariation between regulation and strategy use is most evident in online/hybrid environments (Broadbent, 2017). Accordingly, the present study proposes the following hypothesis (8):

*H8*: Regulating is positively associated with task strategies among Chinese distance learners.

A large body of evidence shows that “task strategies” such as elaboration, organization, repetition, and self-testing are positively correlated with academic performance, and this effect is robust across university samples (Richardson et al., 2012). In online higher education, the contribution of strategy use to academic performance remains significant (Broadbent and Poon, 2015). Accordingly, the present study proposes the following hypothesis (9):

*H9*: Task strategies are significantly associated with academic performance among Chinese distance learners.

## 4 Materials and methods

### 4.1 Methods

This study adopted a quantitative research design to examine how metacognitive regulation relates to academic performance among Chinese distance learners (Ghanad, 2023; Mahat et al., 2024). A total of 381 students enrolled in distance education programs in China completed a survey assessing key aspects of metacognitive regulation. The survey data were analyzed using appropriate statistical techniques to test the proposed relationships and to evaluate the extent to which metacognitive regulation is associated with learners’ academic performance in this context.

### 4.2 Data collection

Primary data were collected via a survey to support quantitative analysis among Chinese distance learners. Collecting data directly from participants allowed the study to capture learners’ current experiences and perceptions relevant to metacognitive regulation and academic performance (Mazhar et al., 2021; Ganesha and Aithal, 2022). This approach enhances the contextual relevance of the evidence and helps ensure that the data reflect the specific distance learning setting examined in the present study.

In China, distance education has expanded rapidly, with the Ministry of Education reporting approximately 8.465 million learners engaged in distance education and 2.779 million enrolled in formal distance education programs (Chen et al., 2024). Accordingly, Chinese distance learners constituted the target population for the present study. Given the large population size, a commonly used sample size benchmark for survey studies indicates that a minimum of 384 responses would provide adequate precision for estimating population parameters. In practice, 381 valid survey responses were obtained, which is very close to the recommended threshold and therefore considered sufficient for the planned analyses. Participant recruitment and data collection were conducted between January 2023 and December 2024, a predefined window aligned with the project timeline and deadline.

The 381 respondents were students enrolled in distance or online learning programs in China, primarily offered by higher education institutions (i.e., universities and university-affiliated continuing or online education units) rather than vocational or trade schools. Eligibility required prior experience taking online or distance learning courses. To enhance sample diversity, participants were recruited through multiple channels, including the distribution of the survey link via Chinese social media platforms (e.g., Xiaohongshu and Weibo) and university-affiliated WeChat student groups. Consequently, the sample includes learners from a range of institutions and distance education programs across China, spanning both undergraduate and postgraduate levels. Because recruitment was conducted through an open online approach, the exact number of institutions represented could not be confirmed; however, using multiple recruitment channels helped capture a heterogeneous group of distance learners with varied academic backgrounds and enrolment durations. Table 1 summarizes the key variables used in this study.

**Table 1:** Variable definition.

Abbreviation	Full term	Description
AP	Academic performance	Dependent variable representing students’ academic outcomes
TS	Task strategies	Mediating variable reflects students’ use of task-based learning strategies
M	Monitoring	Independent variable indicating students’ ability to track and assess their learning
P	Planning	Independent variable reflecting students’ capacity to set goals and organize study activities
R	Regulation	Independent variable representing students’ ability to control and adjust their learning processes
SE	Self-efficacy	Independent variable capturing students’ confidence in their academic abilities

Survey items were adapted from established instruments and administered to Chinese distance learners. To support linguistic equivalence and cultural appropriateness, the questionnaire was translated into Chinese and back-translated into English by bilingual researchers using standard cross-cultural adaptation procedures. Any inconsistencies between the original and back-translated versions were discussed and resolved to strengthen semantic consistency. The Chinese version was then reviewed by domain experts to assess clarity, relevance, and cultural suitability for the distance learning context. A small pilot check was conducted to confirm readability and comprehension, and minor wording revisions were made accordingly. In the main study, the measurement model was evaluated using PLS-SEM, including assessments of internal consistency reliability as well as convergent and discriminant validity.

### 4.3 Online survey

An online survey was administered to collect data from Chinese distance learners. This mode of data collection was suitable because participants were geographically dispersed and could be reached efficiently through online distribution (Mazhar et al., 2021; Taherdoost, 2022). The survey link was disseminated via multiple channels, including Chinese social media platforms. The questionnaire drew on established self-regulated learning measures and was wording-adjusted where necessary to fit the distance learning context. Specifically, self-efficacy was measured using items adapted from the MSLQ self-efficacy subscale (Pintrich et al., 1991). Planning, monitoring, and regulation were measured using items primarily adapted from the Metacognitive Awareness Inventory (Schraw and Dennison, 1994), supplemented by online SRL instruments such as the OSLQ (Barnard et al., 2009) and SRL-O (Broadbent et al., 2023) when better aligned with the construct and learning context. Academic performance was operationalized as self-reported perceived academic performance rather than objective grades; participants evaluated their perceived learning outcomes using items previously applied in online learning research. The established measures have demonstrated acceptable reliability and validity in prior studies. Responses were recorded on a five-point Likert scale (1 = Strongly Disagree to 5 = Strongly Agree), and the resulting data were analyzed using statistical techniques (Russo et al., 2021).

### 4.4 Data analysis

Survey data were analyzed to address the study objectives and test the hypothesized relationships among the constructs. Initial screening was conducted in IBM SPSS, including checks for missing data and descriptive statistics. The main analyses were then carried out using partial least squares structural equation modeling (PLS-SEM) in SmartPLS (Ringle et al., 2015). SmartPLS was used to assess the measurement model, including internal consistency reliability as well as convergent and discriminant validity, and to estimate the structural model by examining path coefficients and explained variance (R^2^). The significance of hypothesized direct effects and indirect (mediating) effects was evaluated using bootstrapping procedures. Together, these analyses provide evidence on the adequacy of the measurement model and the proposed relationships among the predictors, the mediator, and academic performance.

## 5 Results

### 5.1 Participant demographics

The main aim of the demographic profiling test is to identify the background characteristics of the selected respondents. The results of Table 2 represent the rate of participants who participated in the survey.

**Table 2:** Result of survey participants’ background.

Variable	Category	Count	Column *N* %
Age	18 to 24 years	83	21.8%
	25 to 34 years	134	35.2%
	35 to 44 years	102	26.8%
	45 years and above	62	16.3%
Gender	Male	217	57.0%
	Female	164	43.0%
Level of study	Undergraduate	179	47.0%
	Postgraduate	202	53.0%
Enrolment year	Less than 6 months	168	44.1%
	6 months to 1 year	135	35.4%
	1 to 2 years	54	14.2%
	More than 2 years	24	6.3%
Field of study	Science & Technology	186	48.8%
	Business & Economics	120	31.5%
	Social Sciences	62	16.3%
	Humanities & Arts	8	2.1%
	Others	5	1.3%

Table 2 summarizes the respondents’ demographic characteristics. The largest age group was 25–34 years (*n* = 134, 35.2%), whereas the smallest proportion was aged 45 years and above (*n* = 62, 16.3%). In terms of study level, more than half of the participants were postgraduate students (*n* = 202, 53.0%). Nearly half reported a Science and Technology background (*n* = 186, 48.8%). Regarding enrolment duration, 168 respondents (44.1%) had been enrolled for less than six months. Overall, the profile suggests that the sample covers variation in age, study level, disciplinary background, and enrolment duration, supporting a heterogeneous group of Chinese distance learners for subsequent analyses.

### 5.2 Descriptive analysis

The descriptive analysis test assists the first author in determining the mean, standard deviation, and overall central frequency of all variables.

Table 3 summarizes the descriptive statistics. Academic performance has a mean of 3.31 (SD = 0.83), indicating moderate levels of self-reported performance with some variability across respondents. Monitoring shows a similar central tendency and dispersion (*M* = 3.28, SD = 0.88). Among the variables reported, planning has the highest mean (*M* = 3.41, SD = 0.93), suggesting that planning-related behaviors were relatively more frequently endorsed by Chinese distance learners.

**Table 3:** Central frequency of survey items.

Variables	Mean	Standard deviation
Academic performance	3.3141	0.83460
Monitoring	3.2778	0.87637
Planning	3.4059	0.92912
Regulation	3.3320	0.86750
Self-efficacy	3.3443	0.87465
Task strategies	3.2848	0.88789

In addition to the construct-level descriptive statistics reported in Tables 3, 4 presents the item-level rating scores (mean and standard deviation) for all survey indicators. This provides greater transparency regarding the distribution of responses for each measurement item within each construct.

**Table 4:** Item-level rating scores for each construct.

Construct	Item	Item statement	Mean	SD
Self-efficacy	SE1	I believe I will receive an excellent grade in this course.	3.386	1.122
Self-efficacy	SE2	I’m certain I can understand the most difficult material presented in this course.	3.341	1.133
Self-efficacy	SE3	I’m confident I can do an excellent job on the assignments and tests in this course.	3.320	1.150
Self-efficacy	SE4	I’m certain I can master the skills being taught in this course.	3.373	1.173
Self-efficacy	SE5	I am confident I will do well on assignments and projects	3.320	1.160
Self-efficacy	SE6	I believe I can master the knowledge and skills in the course	3.325	1.133
Planning	P1	When my lecturer gives an online task, I analyze it first before starting it	3.378	1.126
Planning	P2	I set some standards (what I need to achieve) for my online task	3.441	1.161
Planning	P3	When reading for this course, I make up questions to evaluate my focus on my reading.	3.425	1.191
Planning	P4	Before thoroughly studying new course material, I often skim it to see how it is organized.	3.472	1.160
Planning	P5	I set realistic deadlines for learning	3.409	1.163
Planning	P6	I plan out my schedule each week, so I have the appropriate amount of time available for online study	3.310	1.268
Monitoring	M1	When doing an online task, I question myself to help me understand the task better	3.289	1.199
Monitoring	M2	When doing an online task, I check my progress with my lecturer to ensure I am on track with my task	3.328	1.218
Monitoring	M3	When I become confused about something I’m reading for this class, I go back and try to figure it	3.283	1.183
Monitoring	M4	If the course materials are difficult to understand, I change the way I read the material.	3.276	1.231
Monitoring	M5	I ask myself questions to make sure I understand the material I have been studying in this class.	3.226	1.221
Monitoring	M6	When I perform tasks, I keep a close track of how well I am doing	3.265	1.214
Regulation	R1	If I get confused during an online task, I use other methods to learn the task	3.270	1.251
Regulation	R2	I check over my online task to make sure I did everything correctly before submitting it.	3.273	1.235
Regulation	R3	I try to think through a topic and decide what I am supposed to learn from it, rather than just reading it over when studying.	3.391	1.171
Regulation	R4	When studying for this course, I try to determine which concepts I do not understand well.	3.354	1.162
Regulation	R5	When I study for this class, I set goals for myself to direct my activities in each study period.	3.320	1.146
Regulation	R6	If I get confused taking notes in class, I make sure I sort it out afterwards.	3.383	1.154
Task strategies	TS1	When studying online, I try to relate the content to what I already know	3.375	1.097
Task strategies	TS2	I try to improve my understanding by doing additional work beyond the core content	3.231	1.198
Task strategies	TS3	I find studying for this online class enjoyable	3.281	1.160
Task strategies	TS4	When studying online, I create my own examples of the content to make it more meaningful	3.228	1.158
Task strategies	TS5	When studying online, I organize my thoughts by making summaries of what I am learning	3.299	1.163
Task strategies	TS6	When learning the online content, I try to develop my own ideas about it	3.294	1.141
Academic performance	AP1	I can apply the content learned in the online learning course.	3.315	1.208
Academic performance	AP2	I am more independent after an online learning course	3.354	1.180
Academic performance	AP3	I am a better thinker after an online learning course.	3.386	1.210
Academic performance	AP4	I can demonstrate to others my hands-on skills learned in an online learning course.	3.320	1.134
Academic performance	AP5	I acquired some useful knowledge by interacting with other students during the online learning course	3.239	1.121
Academic performance	AP6	I acquired some useful knowledge by interacting with my lecturer during the online learning course	3.270	1.198

Item-level descriptive statistics (mean and standard deviation) based on the study sample. Higher scores indicate stronger agreement with the statement.

### 5.3 Reliability

The reliability analysis depicts the consistency of the item that has been selected for the study in gathering responses. A value within the range of 0.7 to 0.9, and AVE is a value above 0.5, indicating that the consistency of the items is appropriate. Additionally, the composite reliability values are also checked to see whether they are valued above 0.7 or not. Satisfaction with these factors indicates the consistency of the survey items.

Table 5 summarizes the reliability and convergent validity results for the study constructs. Cronbach’s alpha values indicate acceptable internal consistency for self-efficacy (*α* = 0.857), planning (*α* = 0.874), regulation (*α* = 0.828), and monitoring (*α* = 0.818), all falling within the commonly accepted 0.70–0.90 range. Task strategies (*α* = 0.861) and academic performance (*α* = 0.804) likewise demonstrate satisfactory internal consistency. Composite reliability values also met recommended criteria, providing further support for scale reliability. In addition, all average variance extracted (AVE) values exceeded 0.50, indicating adequate convergent validity of the measurement model.

**Table 5:** Indicator reliability.

Construct	Cronbach’s alpha	Composite reliability (rho_a)	Composite reliability (rho_c)	Average variance extracted (AVE)
AP	0.804	0.806	0.859	0.505
M	0.818	0.825	0.870	0.532
P	0.874	0.873	0.906	0.618
R	0.828	0.832	0.875	0.542
SE	0.857	0.855	0.895	0.589
TS	0.861	0.860	0.898	0.600

### 5.4 Validity

Discriminant validity was assessed using the heterotrait-monotrait (HTMT) ratio. Following commonly used guidelines, HTMT values of 0.85 or below indicate adequate discriminant validity between constructs, whereas values exceeding this threshold may suggest insufficient construct distinctiveness and potential overlap in the measurement model.

From Table 6, it is evident from the table above that all the values for the respective variables have been obtained to be less than 0.85. This ensures that the student’s validity has been established across all the constructs. None of the pair of variables portray excessive similarity, conveying that each construct appears to be measuring a different concept, which is necessary for the analysis.

**Table 6:** Heterotrait-Monotrait (HTMT) ratio.

Construct	AP	M	P	R	SE	TS
AP						
M	0.746					
P	0.612	0.756				
R	0.614	0.732	0.570			
SE	0.607	0.683	0.661	0.491		
TS	0.611	0.667	0.561	0.661	0.517	

### 5.5 Regression analysis

#### 5.5.1 Correlation analysis

The multiple linear regression aims to figure out the impact of independent variables on the dependent variable. It has been stated that the *p*-value should be 0.05 or less to make the linear relationship between variables significant.

**Figure 3:**
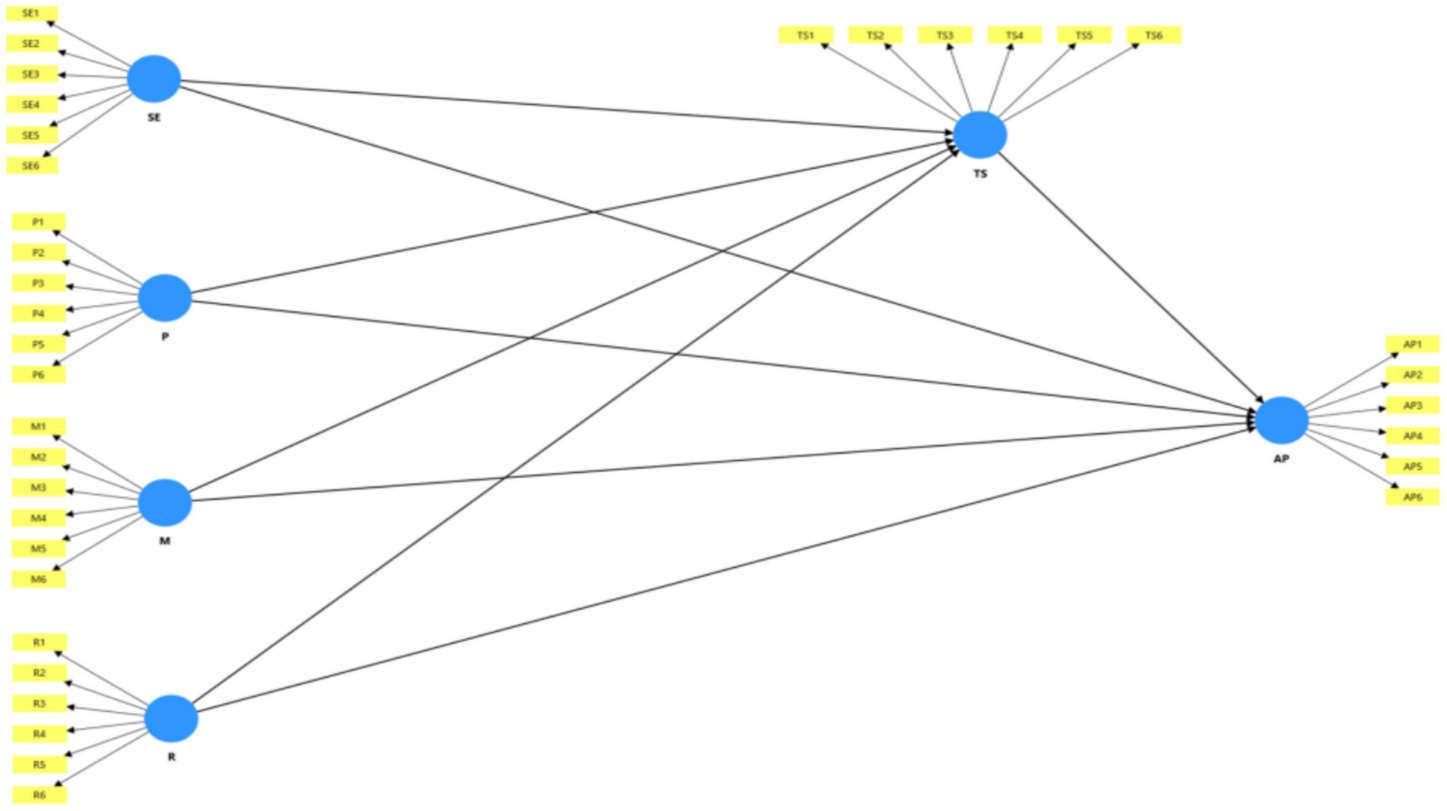
Initial model.

**Figure 4:**
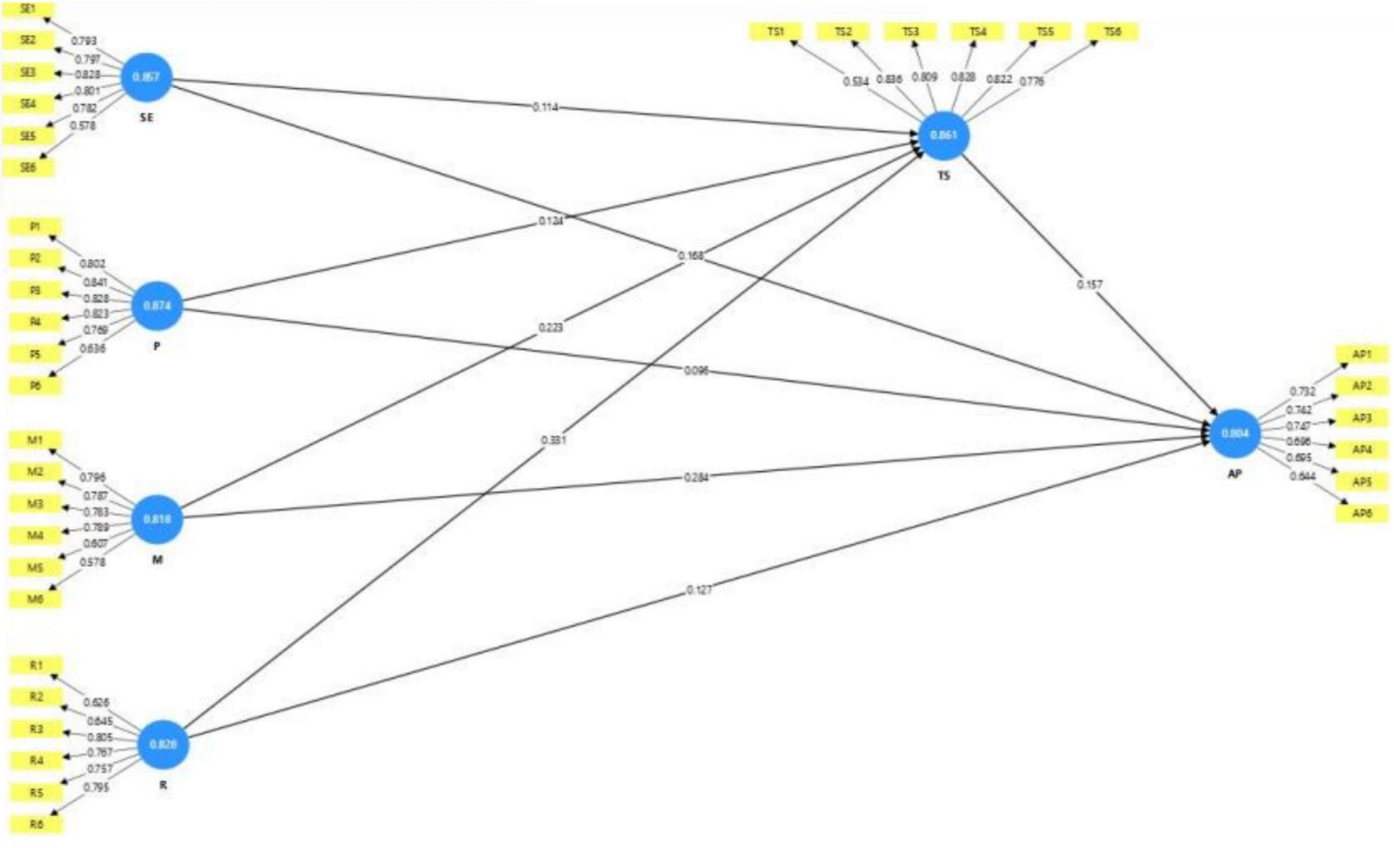
Final model.

Table 7 and Figures 2, 3 summarize the regression results. Overall, the predictors show meaningful associations with academic performance in this sample. Monitoring and regulation exhibit statistically significant positive relationships with academic performance (*p* < 0.05). By contrast, planning provides only marginal evidence of a direct association with academic performance, suggesting a weaker or less proximal link in this model. Taken together, the results indicate differentiated effects across the metacognitive regulation components rather than a uniform pattern (Figure 4).

**Table 7:** Regression analysis.

Path	Original sample (O)	Sample mean (M)	Standard deviation (STDEV)	T statistics (|O/STDEV|)	*p* values
M -> AP	0.284	0.285	0.056	5.101	0.000
M -> TS	0.223	0.225	0.058	3.870	0.000
P -> AP	0.096	0.097	0.050	1.917	0.055
P -> TS	0.124	0.123	0.056	2.207	0.027
R -> AP	0.127	0.128	0.050	2.568	0.010
R -> TS	0.331	0.330	0.048	6.910	0.000
SE -> AP	0.168	0.169	0.050	3.339	0.001
SE -> TS	0.114	0.114	0.051	2.249	0.025
TS -> AP	0.157	0.156	0.052	3.039	0.002

Beyond the regression results reported in Table 7, the structural relationships were further examined using the PLS-SEM model in Table 8. The PLS-SEM estimates largely corroborate the regression patterns and, through bootstrapping, provide confidence intervals for the path coefficients, offering a more detailed basis for interpreting the robustness of the model results.

**Table 8:** Path coefficients from PLS-SEM analysis.

Path	Coefficient (*β*)	*t*-statistic	*p*-value	95% Confidence interval
M → AP	0.284	5.1	0.0000	[0.174, 0.394]
M → TS	0.223	3.87	0.0001	[0.111, 0.339]
P → AP	0.096	1.92	0.0550	[0.000, 0.195]
P → TS	0.124	2.21	0.0270	[0.111, 0.339]
R → AP	0.127	2.57	0.0100	[0.030, 0.225]
R → TS	0.331	6.91	0.0000	[0.235, 0.428]
SE → AP	0.168	3.34	0.0010	[0.070, 0.267]
SE → TS	0.114	2.25	0.0250	[0.015, 0.213]
TS → AP	0.157	3.04	0.0020	[0.056, 0.259]

The results indicate that metacognitive factors, particularly monitoring, regulation, and self-efficacy, are positively associated with academic performance. Task strategies also significantly predict academic performance, supporting their role as predictor in distance education. Planning shows marginal evidence in relation to academic performance, but it is positively associated with task strategies. Overall, the findings suggest that metacognitive regulation and self-efficacy are important correlates of students’ academic performance in this sample.

#### 5.5.2 Mediating effect

In addition to examining the direct effects in the structural model, this study assessed the indirect (mediating) effects of task strategies using bootstrapping procedures in PLS-SEM. The results are shown in Table 9.

**Table 9:** Specific indirect effects from PLS-SEM analysis.

Indirect path	Coefficient (*β*)	*t*-statistic	*p*-value	95% Confidence interval
M → TS → AP	0.0351	2.27	0.023	[0.010, 0.073]
P → TS → AP	0.0195	1.8	0.073	[0.001, 0.043]
R → TS → AP	0.052	2.77	0.006	[0.017, 0.091]
SE → TS → AP	0.0179	1.74	0.081	[0.002, 0.041]

Table 9 shows that the mediating role of task strategies is path-specific rather than uniform across predictors. The indirect effects from monitoring (*β* = 0.0351, *p* = 0.023) and regulation (*β* = 0.0520, *p* = 0.006) to academic performance via task strategies are statistically significant, indicating that task strategies partially carry the effects of these metacognitive processes. By contrast, the corresponding indirect effects for planning (*β* = 0.0195, *p* = 0.073) and self-efficacy (*β* = 0.0179, *p* = 0.081) do not reach conventional significance levels, although both coefficients are positive. Overall, the pattern is consistent with partial and relatively modest mediation, with clearer support for monitoring and regulation than for planning and self-efficacy in this sample.

### 5.6 Additional analysis

#### 5.6.1 Differences by age group

Given the wide age range of the sample, an additional analysis was conducted to examine whether key constructs differed across age groups. Considering the ordinal nature of the Likert-scale data and the unequal sample sizes across groups, the Kruskal-Wallis test was employed to compare self-efficacy, metacognitive regulation (planning, monitoring, and regulation), task strategies, and academic performance across four age groups (18–24, 25–34, 35–44, and 45 years and above). Table 10 presents the mean (SD), sample size for each age group, and the corresponding Kruskal-Wallis test statistics.

**Table 10:** Age-group differences in key constructs (Kruskal-Wallis test).

Construct	18 to 24 years	25 to 34 years	35 to 44 years	45 years and above	Kruskal-Wallis H	*p*-value
Self-efficacy	3.454 (0.803) [*n* = 83]	3.372 (0.913) [*n* = 134]	3.247 (0.829) [*n* = 102]	3.298 (0.952) [*n* = 62]	2.503	0.475
Planning	3.426 (0.926) [*n* = 83]	3.388 (0.967) [*n* = 134]	3.307 (0.839) [*n* = 102]	3.581 (0.933) [*n* = 62]	3.316	0.345
Monitoring	3.353 (0.877) [*n* = 83]	3.310 (0.956) [*n* = 134]	3.136 (0.791) [*n* = 102]	3.341 (0.821) [*n* = 62]	3.365	0.339
Regulation	3.337 (0.880) [*n* = 83]	3.374 (0.896) [*n* = 134]	3.271 (0.844) [*n* = 102]	3.333 (0.840) [*n* = 62]	0.715	0.870
Task strategies	3.215 (0.879) [*n* = 83]	3.358 (0.934) [*n* = 134]	3.255 (0.823) [*n* = 102]	3.269 (0.910) [*n* = 62]	1.535	0.674
Academic performance	3.331 (0.846) [*n* = 83]	3.366 (0.859) [*n* = 134]	3.239 (0.774) [*n* = 102]	3.304 (0.873) [*n* = 62]	1.122	0.772

Cells report Mean (SD) and sample size for each age group. *p*-values are from the Kruskal-Wallis test.

Table 10 indicates that none of the examined constructs differed significantly across age groups (all *p*-values > 0.05). Overall, respondents across the four age categories reported similar levels of self-efficacy, metacognitive regulation (planning, monitoring, and regulation), task strategies, and academic performance. This pattern suggests limited age-related variation in the present dataset and reduces the likelihood that the main PLS-SEM relationships are attributable to systematic differences in learners’ age.

#### 5.6.2 Differences by field of study

To examine whether key constructs differed across students’ fields of study, an additional non-parametric comparison was conducted. Given the ordinal nature of Likert-scale responses and unequal group sizes, Kruskal-Wallis tests were used to compare self-efficacy, metacognitive regulation (planning, monitoring, and regulating), task strategies, and academic performance across three major fields with adequate sample sizes (Science & Technology, Business & Economics, and Social Sciences). Table 11 reports the mean (SD) and sample size for each field, along with the Kruskal-Wallis H statistics and corresponding *p*-values.

**Table 11:** Field-of-study differences in key constructs (Kruskal-Wallis test).

Construct	Science & Technology	Business & Economics	Social Sciences	Kruskal-Wallis H	*p*-value
Self-efficacy	3.370 (0.883) [*n* = 186]	3.296 (0.886) [*n* = 120]	3.360 (0.810) [*n* = 62]	0.508	0.776
Planning	3.471 (0.926) [*n* = 186]	3.383 (0.914) [*n* = 120]	3.258 (0.887) [*n* = 62]	2.267	0.322
Monitoring	3.307 (0.894) [*n* = 186]	3.263 (0.907) [*n* = 120]	3.215 (0.747) [*n* = 62]	0.483	0.785
Regulation	3.349 (0.884) [*n* = 186]	3.356 (0.904) [*n* = 120]	3.172 (0.707) [*n* = 62]	1.438	0.487
Task strategies	3.317 (0.843) [*n* = 186]	3.322 (0.956) [*n* = 120]	3.132 (0.846) [*n* = 62]	1.982	0.371
Academic performance	3.337 (0.864) [*n* = 186]	3.315 (0.866) [*n* = 120]	3.231 (0.648) [*n* = 62]	0.520	0.771

Cells report Mean (SD) and sample size for each field. *p*-values are from the Kruskal-Wallis test. Analyses focus on fields with adequate sample sizes (Science & Technology, Business & Economics, and Social Sciences).

As shown in Table 11, the Kruskal-Wallis tests indicate that no statistically significant differences were observed across fields of study for any of the examined constructs, including self-efficacy, planning, monitoring, regulation, task strategies, and academic performance (all *p*-values > 0.05). These results suggest that students from different academic disciplines reported broadly comparable levels of metacognitive processes, self-efficacy, and academic performance. Accordingly, the main structural relationships identified in the PLS-SEM analysis appear robust across fields of study and are unlikely to be driven by disciplinary differences in the present sample.

### 5.7 Hypothesis testing

The proposed hypotheses were evaluated using the significance tests from the regression, mediation, and PLS-SEM analyses. Paths were interpreted as supported when their estimated effects were statistically significant at conventional levels (e.g., *p* < 0.05), and as not supported when they did not reach this threshold. Importantly, statistical significance was considered alongside the direction and magnitude of the estimated coefficients. Overall, the results provide empirical support for several hypothesized relationships in the conceptual model, while indicating that some pathways are weaker or not statistically distinguishable from zero in the present sample.

It is evident from Table 12 that most hypothesized relationships in the structural model meet the conventional significance criterion (*p* < 0.05). Specifically, self-efficacy, monitoring, and regulation show statistically significant positive associations with academic performance. In contrast, the direct association between planning and academic performance is marginally supported (*p* = 0.055) and therefore should be interpreted with caution.

**Table 12:** Hypothesis testing.

Hypotheses	Results
*H1*: Self-efficacy is positively associated with academic performance among Chinese distance learners.	Supported
*H2*: Planning is positively associated with academic performance among Chinese distance learners.	Marginally Supported
*H3*: Monitoring is positively associated with academic performance among Chinese distance learners.	Supported
*H4*: Regulation is positively associated with academic performance among Chinese distance learners.	Supported
*H5*: Self-efficacy is positively associated with task strategies among Chinese distance learners.	Supported
*H6*: Planning is positively associated with task strategies among Chinese distance learners.	Supported
*H7*: Monitoring is positively associated with task strategies among Chinese distance learners.	Supported
*H8*: Regulation is positively associated with task strategies among Chinese distance learners.	Supported
*H9*: Task strategies are significantly associated with academic performance among Chinese distance learners.	Supported

To further examine the proposed role of task strategies in the model, we assessed whether task strategies are significantly associated with academic performance and whether the independent variables are significantly associated with task strategies. The results indicate that task strategies are significantly associated with academic performance, and planning, monitoring, regulation, and self-efficacy are significantly associated with task strategies (Table 12), supporting the inclusion of task strategies as a key strategic correlation in the model.

Regarding mediation, the bootstrapping results suggest that the indirect effects via task strategies are not uniform in strength across predictors. While the indirect pathways for monitoring and regulating through task strategies are statistically significant, the indirect effects for planning and self-efficacy through task strategies are not statistically significant in the present sample (*p* = 0.073 and *p* = 0.081, respectively). Accordingly, the mediating role of task strategies should be characterized as partial and modest, rather than strong across all hypothesized paths. Overall, H5–H9 are largely supported for the direct paths, whereas the mediation hypotheses receive mixed support.

## 6 Discussion

Overall, the findings suggest that metacognitive regulation relates to academic performance in a differentiated way rather than as a uniform “more is better” pattern. Monitoring and regulation show robust positive associations with academic performance, whereas planning displays only marginal evidence for a direct association (*p* = 0.055), indicating that planning may be beneficial but less proximally tied to performance outcomes in this sample. Conceptually, this pattern aligns with Flavell’s (1979) monitoring-control account, in which monitoring provides diagnostic information about understanding and progress and prompts control decisions (e.g., revising strategies, reallocating time, or increasing effort). In addition, the mediating role of task strategies appears path-specific: the indirect effects from monitoring and regulation to academic performance via task strategies are statistically significant, but the corresponding indirect effects for planning and self-efficacy are not (*p* = 0.073 and *p* = 0.081). From a motivational perspective, self-efficacy may still matter because efficacy beliefs influence whether learners initiate and persist in regulatory behaviors, positioning self-efficacy as an important driver of metacognitive strategy enactment (Schunk, 1989; Schunk, 1991). This interpretation is consistent with evidence that metacognition is closely linked with self-efficacy (Celik, 2022) and that distance learners’ online familiarity and attitudes can shape their efficacy beliefs (De Backer et al., 2022). Taken together, these results are most consistent with a partial-process account in which performance benefits arise primarily when learners detect mismatches during learning (monitoring) and subsequently adapt their approach (regulation), with task strategies transmitting part but not all these effects.

The contrast between planning and monitoring is theoretically informative. Planning may reflect learners’ intentions and preparatory organization, but in distance learning it does not necessarily translate into effective execution unless it is accompanied by ongoing self-checks and adjustments. This pattern is reflected in the present model: planning shows only marginal evidence for a direct link with academic performance (*p* = 0.055), whereas monitoring demonstrates a clearer association with performance. One plausible interpretation is that monitoring provides the diagnostic feedback learners need to notice gaps in understanding and to decide when to revise strategies, particularly in settings with limited immediate instructor support, as emphasized in monitoring-control accounts of metacognition (Flavell, 1979; Van Loon and Oeri, 2023). A reasonable alternative interpretation is that planning may operate more indirectly, such as through improved strategy selection or time allocation, and therefore appears weaker in a cross-sectional model focusing on perceived performance at a single time point (Roberts, 2021).

The mediation results further refine the mechanism. Task strategies partially transmit the effects of metacognitive regulation, but the mediation is not uniform across predictors: the indirect pathways from monitoring and regulation to academic performance via task strategies are significant, whereas the indirect effects for planning and self-efficacy are not statistically significant at the 0.05 level. This pattern is consistent with metacognitive monitoring-control accounts, which propose that monitoring informs control decisions that are enacted through strategy adjustment and task management (Flavell, 1979), and with evidence that monitoring supports reflective evaluation and adaptive regulation in online and distance learning contexts (Taouki et al., 2022; Van Loon and Oeri, 2023). It also suggests that task strategies may be especially important as an “implementation channel” once learners engage in real-time monitoring and regulation, while planning and self-efficacy may influence performance through additional routes not fully captured by task-strategy indicators (e.g., persistence, help-seeking, or effort regulation), as motivational accounts of self-efficacy emphasize its role in sustaining effort and strategy enactment (Schunk, 1989; Schunk, 1991; Celik, 2022).

## 7 Conclusion and future directions

This study examined how metacognitive regulation relates to academic performance among Chinese distance learners using survey data. Overall, the findings suggest that metacognitive regulation is associated with academic performance in a differentiated manner rather than as a uniform “more is better” pattern. In particular, self-efficacy, monitoring, and regulation show clearer positive relationships with academic performance, whereas planning appears comparatively weaker in its direct link with performance. Taken together, the results highlight the importance of both motivational beliefs (self-efficacy) and performance-phase processes (monitoring and regulation) in supporting learners’ academic outcomes in distance learning contexts.

The analysis also clarifies the role of task strategies in the model. Task strategies are positively related to academic performance and are associated with the key predictors, indicating that they function as an important pathway through which metacognitive and motivational factors may translate into performance-related outcomes. At the same time, the mediation evidence is not uniform across predictors, suggesting that task strategies partially transmit some effects (more clearly for monitoring and regulation) while other influences may operate through additional routes not fully captured in the present model.

From a practical perspective, the findings offer implications for the design of distance learning curricula and learner support. For policymakers and program designers, the results point to the value of embedding structured supports that strengthen learners’ self-efficacy and foster monitoring and regulatory adjustment during learning (e.g., prompts for progress checking, guided reflection, and strategy adaptation). For instructors and learning designers, task structures that encourage purposeful strategy use may help learners remain goal-directed and responsive to learning difficulties in online and distance settings.

Several limitations should be acknowledged. First, the study relies on a cross-sectional survey design, which constrains causal interpretation of the observed relationships. Second, academic performance was measured via self-reported perceived performance rather than objective academic records (e.g., GPA or official course grades), which may introduce self-report bias and increase the risk of common method variance. Third, although the sample draws from multiple programs and recruitment channels within China, the findings should be generalized cautiously to other contexts or populations.

Future research could extend this work in several ways. Replication studies using objective performance indicators (course grades, GPA, or LMS-based assessment scores) would strengthen measurement validity and improve confidence in the model’s conclusions. Longitudinal or time-lagged designs could further clarify the temporal ordering among self-efficacy, metacognitive regulation, task strategies, and performance. In addition, qualitative approaches (e.g., semi-structured interviews with learners and/or instructors) could provide richer insight into how monitoring and regulation unfold in practice and how institutional or instructional conditions shape learners’ strategic behavior in distance learning environments.

## Data Availability

The data analyzed in this study is subject to the following licenses/restrictions: the data is available on request. Requests to access these datasets should be directed to P120538@siswa.ukm.edu.my.
